# Management of Necrotizing Fasciitis and Fecal Peritonitis following Ostomy Necrosis and Detachment by Using NPT and Flexi-Seal

**DOI:** 10.1155/2015/231450

**Published:** 2015-09-10

**Authors:** Fahri Yetışır, Akgün Ebru Şarer, H. Zafer Acar

**Affiliations:** ^1^General Surgery Department, Atatürk Research and Training Hospital, 06800 Ankara, Turkey; ^2^Anesthesiology and Reanimation Department, Atatürk Research and Training Hospital, 06800 Ankara, Turkey; ^3^General Surgery Department, Lokman Hekim Private Hospital, 06100 Ankara, Turkey

## Abstract

Management of necrotizing fasciitis and severe faecal peritonitis following ostomy in elderly patient with comorbid disease is challenging. We would like to report management of frozen Open Abdomen (OA) with colonic fistula following ostomy necrosis and detachment in an elderly patient with comorbid disease and malignancy. 78-year-old woman with high stage rectum carcinoma was admitted to emergency department and underwent operation for severe peritonitis and sigmoid colonic perforation. Loop sigmoidostomy was performed. At postoperative 15th day, she was transferred to our clinic with necrotizing fasciitis and severe faecal peritonitis due to ostomy necrosis and detachment. Enteric effluent was removed from the OA wound by using the Flexi-Seal Fecal Management System (FMS) (ConvaTec) and pesser tube in deeply located colonic fistula in conjunction with Negative Pressure Therapy (NPT). Maturation of ostomy was facilitated by using second NPT on ostomy side. After source control, delayed abdominal closure was achieved by skin flap approximation.

## 1. Introduction

More than half of all patients with intestinal stomas have complications. Complications are classified as early or late. Early complications present immediately after surgery (under a month). Ostomy necrosis and mucocutaneous dehiscence are very important early complications; they may cause major morbidity and even mortality. After ostomy, 7–25% mucocutaneous dehiscence and 1–34% necrosis of ostomy are reported [1]. It may be limited to one part of the ostomy or to total. If necrosis extends below the myofascial and peritoneal layers, immediate reoperation is required for stoma resection and reconstruction [1]. Nursing care is essential for keeping the subcutaneous site clean between the stoma and the skin, filling it with absorbent materials, paste, or powder depending on its depth until the new mucocutaneous junction has healed by secondary intention. In more complex cases, assisted vacuum cures have been used [2].

Open Abdomen (OA) management is a life-saving and challenging strategy in severe generalized peritonitis [3, 4]. Mortality rates up to 50% were reported and even higher in the infected OA [5]. Enteric fistulas are one of the most devastating abdominal complications in abdominal surgery [6]. Management of OA with fistula is very challenging.

Flexi-Seal Fecal Management System (FMS) (ConvaTec) is a device which is used for bedridden or immobilized, incontinent patients with liquid or semiliquid stool. It is designed to safely and effectively contain and divert fecal matter, protect patients' wounds from fecal contamination, and reduce both the risk of skin breakdown and the spread of infection [7].

We would like to report treatment of necrotizing fasciitis and fecal peritonitis following ostomy necrosis and detachment by OA management with NPT. We would like also to emphasize, in this case, converting fistula to ostomy by the help of Flexi-Seal FMS and second NPT.

## 2. Presentation of Case

78-year-old woman with high stage rectum carcinoma was planned to take neoadjuvant chemoradiotherapy for down staging. During this period, she was admitted to emergency department with complaint of abdominal pain, distention, constipation, and vomiting. For last 3 days, the severity of complaints has increased. Her consciousness and orientation were also worsened. In her past history, she had hypertension, diabetes mellitus (DM), and hyperlipidemia. In physical examination, vital parameters were as follows: blood pressure (BP): 120/65 mmHg, heart rate (HR): 86, respiratory rate (RR): 24, fever (F): 38°C, and body mass index (BMI): 18. On her abdominal examination there was distention on abdomen and hyperactive bowel sounds were present. Rebound and rigidity were positive at all quadrants of abdomen. Air/fluid level was seen on abdominal X-ray film. She underwent emergent operation. At first operation, severe peritonitis and perforation of dilated sigmoid colon were seen at exploration. Curative oncological resection could not be applied, operation had to be deliberately abbreviated due to hemodynamic instability and physiologic derangement of patient, and so septic abdomen was irrigated, and loop sigmoidostomy was performed. At postoperative 7th day, there was infective fluid coming from midline incision. After drainage and debridement, local NPT was applied to incision.

**Figure 1:**
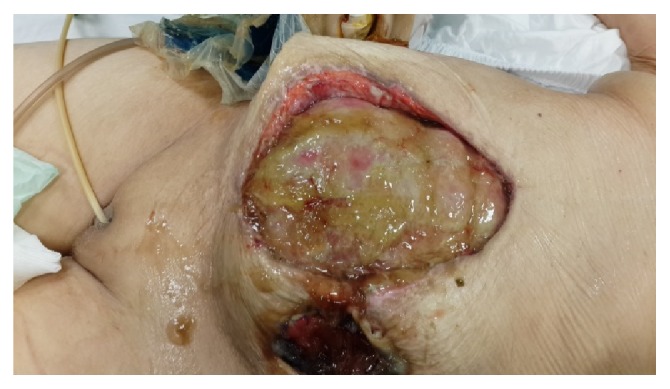
Edematous and fragile bowel with enteric fistula is seen in OA. Communication between ostomy opening and OA. Separation of ostomy from the skin and division of proximal and distal part of sigmoid colon after ostomy necrosis are seen.

**Figure 2:**
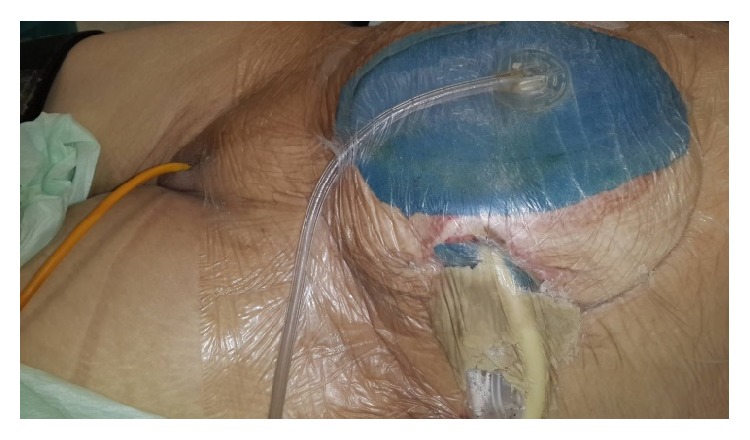
Glycerin-impregnated gauze ostomy opening, pesser drainage tube, and Flexi-Seal with applied abdominal NPT are seen.

At postoperative 15th day, ostomy necrosis was identified and the patient was consulted to our clinic. Her general condition, consciousness, and orientation were not well. She was mechanically ventilated. Her vital parameters were BP: 85/50 mmHg, HR: 120, RR: 28, F: 38°C, and intra-abdominal pressure (IAP): 14 mmHg. She was in septic shock and mild acidosis (Table 1). SOFA score of the patient was 12 and estimated mortality was 50% accordingly. She underwent emergent reoperation. Necrosis all around the ostomy and complete collapse with mucocutaneous detachment of ostomy were seen. Connection was present between the ostomy opening and midline incision with severe fasciitis and fecal peritonitis. Detached proximal and distal sigmoidostomy openings were seen in abdomen deeply (Figure 1). According to new modified Björck classification OA score of the patient was 4 [8]. All intra-abdominal content was irrigated with saline. Since bowel was very edematous and fragile with severe adhesion and short mesentery, these openings of ostomies could not be mobilized. New proximal end transvers colostomy was opened on right side hardly. To redirect colonic effluent to outside, Flexi-Seal was inserted into proximal opening of detached sigmoidostomy and pesser tube was inserted into distal opening. Glycerin-impregnated gauze was used around detached ostomy opening; pesser drainage tube and Flexi-Seal were also used to support segmentation of ostomy side from OA wound (Figure 2). Abdominal NPT was applied to OA. Low dose enteral nutrition was started at postoperative 1st day and increased day by day.

**Figure 3:**
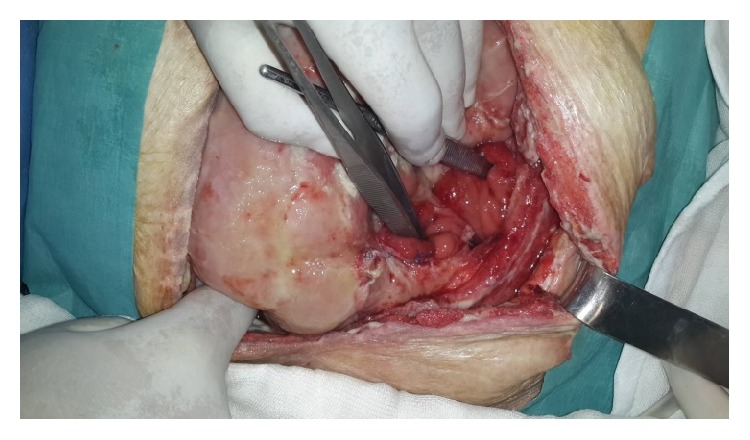
Seperated proximal and distal colonic ends are located deeply in frozen abdomen.

**Figure 4:**
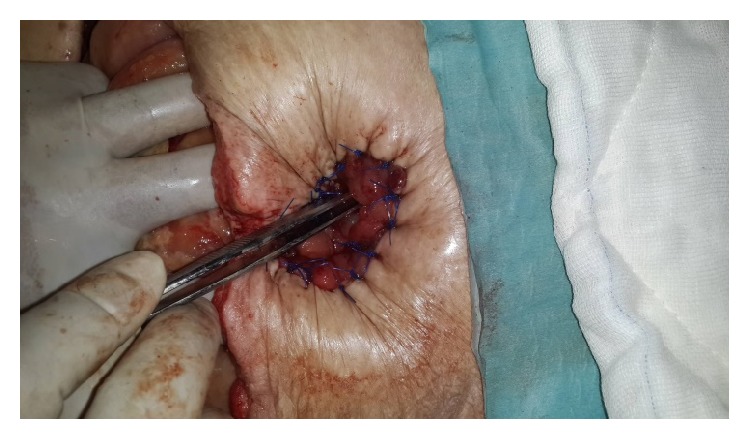
Performed ostomy by inverting skin is seen.

**Figure 5:**
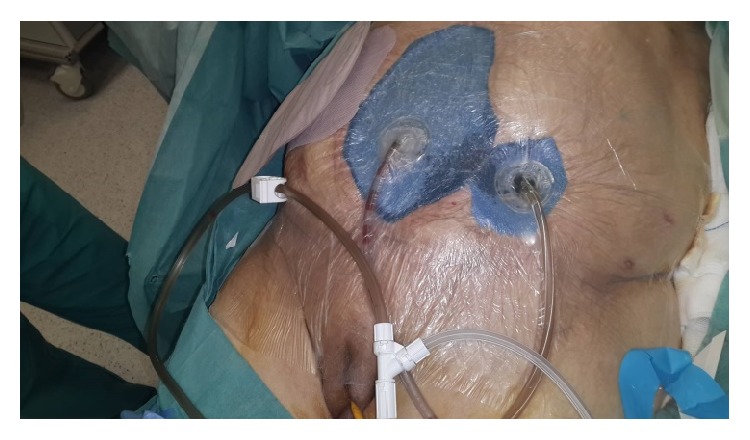
Two NPT are seen; one is abdominal NPT and second one is NPT applied on ostomy.

At postoperative 18th day, Flexi-Seal and pesser tube were removed from the proximal and distal colonic ends (Figure 3). Posterior parts (mesenteric side) of ostomy opening were sutured to each other to make one opening. This opening was converted to ostomy by inverting skin (Figure 4) Glycerin-impregnated gauze and ostomy paste was used over this ostomy. Two NPT systems were applied; one was standard abdominal NPT; second one was performed on the newly created ostomy. Synchronized negative pressure was applied to both of NPTs (Figure 5) [9]. The second NPT on ostomy place was changed 3-4 times a day. Abdominal NPT was changed at 2–4 days' interval. Three days later, stoma maturation completed and second NPT application was stopped. Stoma bags could be applied.

**Figure 6:**
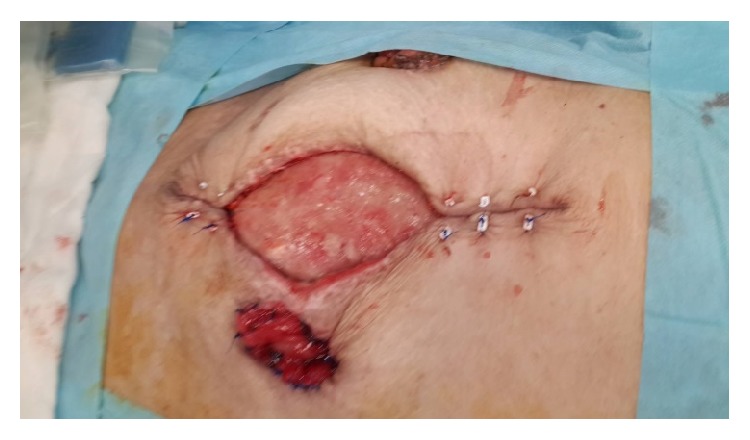
After source control was achieved, good granulation occurred and started to close step by step.

**Figure 7:**
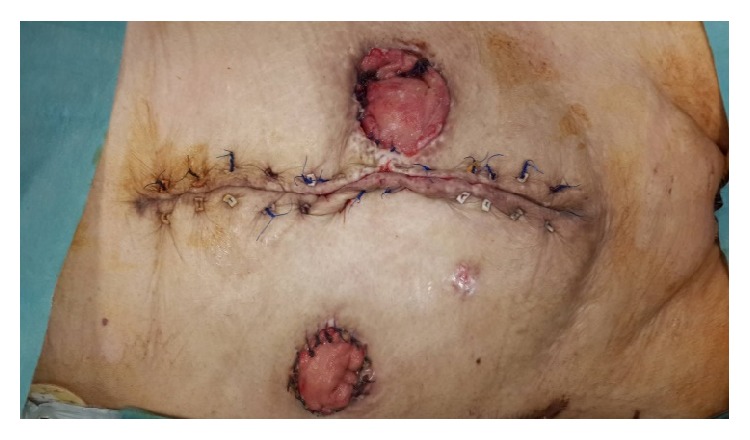
Completed closure is seen.

At postoperative 23rd day, source control was achieved with two ostomies and OA wound was starting to close (Figure 6). At postoperative 38th day all OA was closed (Figure 7).

## 3. Discussion

Severe necrotizing fasciitis and fecal peritonitis due to ostomy necrosis and detachment are a rare but debating complication [1]. Predisposing factors for stoma complication are age, inflammatory bowel disease, body mass index, comorbidity, diabetes, ASA, lack of preoperative care by stoma nurse specialists, and emergent surgery. Many of these factors cannot be controlled by the surgeon but it is essential to consider that many complications are linked with the surgical technique and therefore can be prevented. In our case, an elderly patient with comorbid disease and high stage malignancy had to undergo emergent surgery. If correct diagnosis and proper treatment of ostomy complication are made timely, it can be treated with a very little cost and effort without morbidity and mortality [1].

There are lots of different systems for controlling of EAF but controlling the fistula in frozen abdomen, especially deeply localized one, is very difficult [10]. Tube VAC technique can be used for this kind of patient. The EAFs were intubated using Malecot catheters of appropriate sizes, while the surface of the OA was covered by petroleum-impregnated gauzes and on top by a polyurethane sponge, through which the Malecot catheters were removed [11]. The Flexi-Seal device offers another alternative for effluent control. It is useful regardless of whether the enteric fistulas are low or high output and can even be useful in patients not on bowel rest. Hardwicke showed the successful continent diversion of gastrointestinal secretions in patients with EAF by the Flexi-Seal [12]. Salgado et al. reported that the Flexi-Seal device serves as a valuable tool in aiding in effluent control in complex abdominal wall reconstruction in patients presenting with EAF [13]. Our EAF controlling system was similar to this system in some aspects. In our case, enteric effluent was removed from OA wound by using Flexi-Seal in deeply localized fistula in conjunction with NPT. This procedure may gain time to patient as well as to surgeon until adequate source control could be achieved. Three days after this operation, intra-abdominal sepsis and edema were resolved and these freely located intra-abdominal two colonic ends could be converted into one ostomy.

For stoma formation, good release of the segment next to the intestine is required, for tension-free exteriorization [14]. In obese and/or in patients with short mesentery these procedures may be more difficult. Technical modifications have been described in patients with short mesenteries, such as the suture-bridge or subcutaneous bridge device [15]. Therefore, to minimize risk of retraction, an end loop colostomy has been described where the end of the closed colon remains inside the abdomen and the antimesenteric side is open as a loop colostomy [1]. In our case, there were two major gains by suturing mesenteric side of intra-abdominal two free ends of sigmoidostomy. One of them was converting of the two free intra-abdominal colonic ends into one opening. Second one is to facilitate the degree of retraction due to short mesentery while making ostomy as the same mind of end loop ostomy.

There are lots of different delayed OA closure methods with NPT. One of them was skin flap approximation. This type of closure is more suitable, especially for elderly patients with comorbid disease as in our case. If only skin closure without fascial closure for delayed OA closure is performed, treatment can be terminated with less number of operations in a shorter time period.

## 4. Conclusion

For deeply localized fistula by using Flexi-Seal in conjunction with NPT, enteric effluent can be removed from OA wound. For a short time second NPT application on ostomy may facilitate ostomy maturation. Delayed abdominal closure with skin flap approximation is suitable in infected OA, especially for elderly patients with comorbid disease.

## Figures and Tables

**Table 1:** Laboratory value of patients during consultation.

Biochemical analysis		Total blood count				Blood gas	
Glu	234 mg/dL	WBC	18.000 K/*μ*L	CRP	21 mg/dL (0–0.8)	pH	7.31
K	3.4 mmol/L	Hb	9.8 g/dL	INR	1.7	pCO_2_	38
Alb	1.8 g/dL	Plt	311 K/*μ*L	Procalcitonin	17 ng/mL (<0.500)	PO_2_	73
LDH	211 U/L					HCO_3_	16
T. bilirubin	3.4 mg/dL					BE	−8
Creatinin	2.1 mg/dL						
Urea	87 mg/dL						
